# Modeling Circadian Phototransduction: Retinal Neurophysiology and Neuroanatomy

**DOI:** 10.3389/fnins.2020.615305

**Published:** 2021-02-05

**Authors:** Mark S. Rea, Rohan Nagare, Mariana G. Figueiro

**Affiliations:** ^1^Lighting Research Center, Rensselaer Polytechnic Institute, Troy, NY, United States; ^2^Icahn School of Medicine, Mount Sinai, New York, NY, United States

**Keywords:** circadian phototransduction, shunting inhibition, photic sub-additivity, retinal neurophysiology, retinal neuroanatomy

## Abstract

The retina is a complex, but well-organized neural structure that converts optical radiation into neural signals that convey photic information to a wide variety of brain structures. The present paper is concerned with the neural circuits underlying phototransduction for the central pacemaker of the human circadian system. The proposed neural framework adheres to orthodox retinal neuroanatomy and neurophysiology. Several postulated mechanisms are also offered to account for the high threshold and for the subadditive response to polychromatic light exhibited by the human circadian phototransduction circuit. A companion paper, modeling circadian phototransduction: Quantitative predictions of psychophysical data, provides a computational model for predicting psychophysical data associated with nocturnal melatonin suppression while staying within the constraints of the neurophysiology and neuroanatomy offered here.

## Introduction

In humans, like all other mammals and many other species, the orchestration of physiology and behavior to the natural light-dark cycle is governed by a tight neural coupling of the phototransduction mechanisms in the retina with the master clock in the brain. Indeed, the retino-hypothalamic tract (RHT) has been identified as the neural pathway directly linking the retina to the master clock in suprachiasmatic nuclei (SCN) (Hannibal, 2002), and this neural channel is distinct from those pathways in the optic nerve linking the retina to the major relay center in the brain for vision, the lateral geniculate nuclei (LGN) (De Valois et al., 1966; Hubel, 1988; Hendry and Reid, 2000).

Although the optic nerve carries both visual and non-visual signals to the brain, neural signals carried by both channels originate from the same photoreceptors, of which there are three classes; rods, cones, and intrinsically photosensitive retinal ganglion cells (ipRGCs). Over the past century much has been learned about these photoreceptors and how they initiate neural signals from optical radiation incident on the retina. It is well known now that one of five photopigments is contained within one of five distinct types of photoreceptors. Rods, which are the most common photoreceptor, contain the photopigment rhodopsin (λ_*max*_ ≈ 498 nm) (Hecht et al., 1942). The ipRGCs (Berson et al., 2002) contain OPN4 (melanopsin, λ_*max*_ ≈ 480 nm) (Provencio et al., 1998). The three cones types, long-wavelength (L-) sensitive, middle-wavelength (M-) sensitive and short-wavelength (S-) sensitive, contain OPN1LW (erythrolabe, λ_*max*_ ≈ 565 nm), OPN1MW (chlorolabe, λ_*max*_ ≈ 535 nm), and OPN1SW (cyanolabe, λ_*max*_ ≈ 430 nm), respectively (Smith and Pokorny, 1975).

Signals from these pre-retinal filtered (largely by the crystalline lens; Wyszecki and Stiles, 1982) photoreceptors initiate neural signals that are processed by complex feedforward and feedback neural mechanisms in the retina. Broadly, there are two types of pathways in the retina. Direct pathways transmit the photoreceptor responses either through an amacrine-bipolar neuron complex (rods) or straight through the bipolar neurons (cones) to the ganglion cells, the axons of which form the optic nerve or the RHT. Lateral pathways excite or inhibit activities in adjacent and more distant direct pathways through horizontal and amacrine cells. Importantly, these various direct and lateral neurons perform a variety computations so that by the time the neural signals initiated by the photoreceptors leave the retina via RGC axons, they have been highly processed into different types of information that will, in turn, be interpreted by different centers in the brain (Livingstone and Hubel, 1988; Field et al., 2007, 2009; Erskine and Herrera, 2014; Rea, 2018).

In 2005 the first model of circadian phototransduction was published based upon psychophysical studies relating optical radiation on the retina and its effect on suppressing the synthesis of melatonin by the pineal gland (Rea et al., 2005). Two minor revisions were published in 2012 (Rea et al., 2012) and in 2018 (Rea and Figueiro, 2018). The 2005 model and subsequent revisions were constrained by orthodox retinal neuroanatomy, taking into account published photoreceptor action spectra (e.g., Smith and Pokorny, 1975) and documented pre-retinal filtering (Wyszecki and Stiles, 1982) together with well-established principles of retinal neurophysiology (Kolb et al., 2004). The model was able to predict nocturnal melatonin suppression from both monochromatic and polychromatic light spectra of different amounts without having to evoke *post hoc* fitting functions unrelated to retinal neurophysiology and neuroanatomy. Since the publication of the model in 2005, however, new insights into the neurophysiology of the retina have emerged. For example, *en passant* synapses between S-cone bipolar (SB) neuron and the M1 ipRGC in the most distal, OFF, sublayer of the inner plexiform layer (IPL) of the retina have now been identified (Dumitrescu et al., 2009; Patterson et al., 2020). The purpose of the present paper is to provide an updated physiological foundation for the revised model of circadian phototransduction circuit discussed in Rea et al. (2021).

## Neurophysiology and Neuroanatomy of Circadian Phototransduction

**FIGURE 1:**
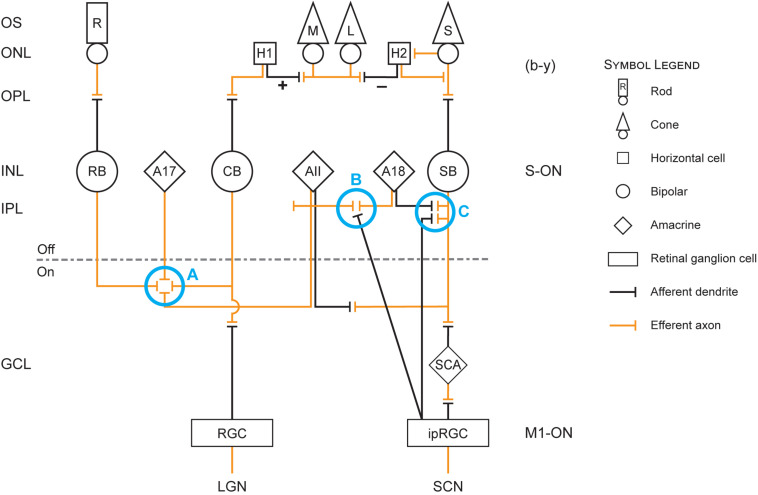
Retinal circuit diagram illustrating the revised model of circadian phototransduction. On the left side of the figure are the conventional labels for the different layers of the retina: OS, outer segment of the rod (R) and cone [L (long-wavelength sensitive), M (middle-wavelength sensitive), and S (short-wavelength sensitive)] photoreceptors; ONL, outer nuclear layer containing the cell bodies of the rod and cone photoreceptors and two types of horizontal cells (H1 and H2); OPL, outer plexiform layer containing the distal plexus of the photoreceptor (efferent, orange lines) axons and the (afferent, black lines) dendrites of the horizontal and bipolar neurons; INL, inner nuclear layer containing the cell bodies of the rod bipolar (RB) and two cone bipolar neurons, one achromatic cone bipolar (CB) and one S-cone bipolar (SB) neuron and amacrine neurons (AII, A17, A18); IPL, inner plexiform layer containing the plexus of axons and dendrites of the bipolar, amacrine, and ganglion neurons, which is divided into OFF and ON sublayers; GCL, ganglion cell layer containing the cell bodies of the conventional retinal ganglion cells (RGCs) and the intrinsically photosensitive retinal ganglion cells (ipRGCs) as well as a displaced S-cone amacrine (SCA). At the bottom of the figure are the two targets for ganglion cell axons, the RGC axons reaching the lateral geniculate nucleus (LGN) and the ipRGC axons reaching the suprachiasmatic nucleus (SCN). Also shown in the diagram are blue circles (Processes A–C) that represent important processes in the revised model. Process A represents cone inhibition of rods and thereby a reduction in shunting inhibition of the ipRGC by AII amacrine cells in process B. Process B also includes decoupling of shunting inhibition of AII amacrine cells via the A18 amacrine cells when the spectrally opponent SB signals “yellow.” Process C represents the *en passant* complex involving the SB, ipRGC, and A18 neurons.

Figure 1 is useful for visualizing the following discussion of the neuroanatomy and neurophysiology underlying the modeled circadian phototransduction circuit.

### The ipRGC Neurons and the RHT as an Axonal Conduit to the SCN

There are several types of ipRGCs in the human retina, each distinct in their morphology, representing less than 1% of all RGCs (Hannibal et al., 2017; Sondereker et al., 2020). The M1 type is the lynch pin between the light-sensitive retina and the SCN, the biological clock in the hypothalamus that orchestrates our physiology and behavior to the natural 24 h, light dark cycle. Like all RGCs, most M1 cell bodies are in the ganglion cell layer (GCL). Unlike most ON, depolarizing RGCs, however, ipRGCs have an afferent dendritic tree extending to the most distal OFF sublayer (S1) in the inner plexiform layer (IPL) (Dumitrescu et al., 2009; Kim et al., 2012). The efferent axons of the M1 form the retino-hypothalamic tract (RHT) that synapses directly with the ventro-dorsal core of the SCN (Meijer and Schwartz, 2003). There is also a sub-class of M1 ipRGC neurons that have cell bodies displaced (M1d) into the inner nuclear layer (INL) but like the conventional M1 ipRGCs, these M1d neurons have dendrites in S1, the outermost sublayer of the IPL (Patterson et al., 2020). The different roles of the M1 and the M1d ipRGCs in circadian phototransduction are not well understood, but they both send axons through the RHT (Sondereker et al., 2020). For this reason, it will be assumed for modeling purposes that they have similar functions for circadian phototransduction.

The M1 and M1d ipRGCs generate a depolarizing, ON response to photon absorption by its photopigment, melanopsin (Provencio et al., 1998; Graham and Wong, 2016). These ipRGCs also carry ON, depolarizing responses from the more distal neurons, bipolar, and amacrine cells, that themselves have been stimulated by photon absorptions in the rod and cone photoreceptors (Matynia et al., 2012; Mure et al., 2019). Through genetic studies, elimination of the photopigment melanopsin from the ipRGCs does not prevent ON responses initiated by the distal photoreceptors through the bipolar and amacrine cells from reaching the SCN, albeit less strongly (Ruby et al., 2002; Hattar et al., 2003). Thus, processed ON photoreceptor responses can reach the SCN via the RHT. Importantly, OFF hyperpolarizing responses are also generated by the post-photoreceptor neurons in the retina, but the M1 ipRGC does not process this information; M1 ipRGCs respond only to and only conduct post-photoreceptor ON responses (Dacey et al., 2005; Zhao et al., 2014; Graham and Wong, 2016). Also, it should be noted that, unlike most RGCs that have an antagonistic small-center-and-large-surround receptive field organization (either ON-center and OFF-surround or OFF-center and ON-surround), the M1 ipRGCs only exhibit a large, ON-center response (Graham and Wong, 2016). In contrast, the M2 ipRGC does reflect center surround organization (Zhao et al., 2014), but its primary input is to the LGN, not to the SCN (Crook et al., 2009).

### The S-Cone Bipolar (SB), Spectral Opponency, and Subadditivity

One of the fundamental tenets of retinal neurophysiology is that the IPL is segregated into proximal-ON and distal-OFF sublayers. Connections between pairs of bipolar and ganglion cells neurons with mismatched polarity have never been observed (Nelson and Connaughton, 2012). Thus, parallel and distinct ON and OFF pathways exit the eye. M1 ipRGCs appear to partially violate this orthodoxy (Nelson and Connaughton, 2012). Although M1 ipRGCs only exhibit ON responses to a light stimulus, they have dendritic trees throughout the IPL, terminating in the OFF sublayer. It was recently shown that ON bipolar neurons form, what are termed *en passant* synapses with the M1 dendrites in the most distal stratification (S1) of the OFF sublayer (Dumitrescu et al., 2009; Grünert et al., 2011; Kim et al., 2012). Thus, the ON-bipolar cells communicate directly with the ON-ipRGCs through this unusual synaptic connection, thereby maintaining an ON pathway through the OFF sublayer (Process C in Figure 1).

There are probably several types of short-wavelength sensitive bipolar neurons, two of which are of particular interest with regard to the ipRGCs, the SB (Figure 1) and the small bistratified bipolar (not shown in Figure 1). These two types of neurons are morphologically distinct. The SB has a dendritic arbor in the proximal, ON sublayer of the IPL whereas the small bistratified bipolar has dendritic arbors in both the ON and OFF sublayers. Both of these cells exhibit blue-ON and yellow-OFF responses, but the formation of the spectral opponent, blue vs. yellow (b − y), information originates from different mechanisms. Further, these two short-wavelength sensitive bipolar neurons appear to have different functions. The small bistratified bipolar exhibits spectral opponent properties through a direct S-cone input (blue) and indirectly via a connection with an amacrine cell in the OFF sublayer which receives input (yellow) from both L- and M-cone bipolar neurons. The color-coded small bistratified bipolar then synapses with a small bistratified ganglion cell which, in turn projects to the LGN in the brain, the major relay station to the visual cortex (e.g., Field et al., 2009). In contrast, spectral opponency in the SB forms more distally through a specialized horizontal cell (H2) that feeds back information from L- and M-cones on to the S-cone. These H2 horizontal cells only contact cones and are spectrally opponent (Perlman et al., 1995). H2 horizontal cells feedback information from the L- and M-cones on to the S-cone, thereby making the S-cone itself spectrally opponent (Packer et al., 2010) as well as the subsequent SB. The H2 horizontal cell illustrated in Figure 1 depicts the summation (+) of the L- and M-cones and its spectral opposition (−) to the S-cone. It is postulated here that the spectrally opponent SB neurons communicate with the M1 ipRGCs as well as with an unidentified dopaminergic amacrine neuron through the *en passant* synapses (Dumitrescu et al., 2009; Kim et al., 2012).

This synaptic connection between the SB and the M1 ipRGC suggests two important inferences, each supported by the physiological and psychophysical data. First, the SB generates an ON response that can stimulate and, thereby, add to the M1 ipRGC direct response to light. Thus, the spectral sensitivity of the SCN will be broader than any one photopigment in response to short wavelengths (i.e., both S-cone photopigment, OPN1SW, and ipRGC photopigment, OPN4 respond to short wavelengths). Second, because of the inherent spectral opponent nature of the SB, the S-ON input to the M1 ipRGC cannot be predicted from the simple addition of photoreceptor responses to polychromatic light. Rather, in spectral opponent neurons like the SB, photoreceptor responses are differenced, evoking what is known as a *subadditive* response to polychromatic light (Figueiro et al., 2008). For a “white” spectrum that perfectly balances short with long wavelengths for the SB, it will not respond at any light level as long as the balance between short and long wavelengths is preserved. Thus, for a perfectly balanced “white” light source only the intrinsic response of the ipRGC would stimulate the SCN once its response is above threshold.

### The AII Amacrine

The AII amacrine has been called the “rod amacrine” because the magnitude of its response to light stimulation is greatest under scotopic (rod) conditions (Farsaii and Connaughton, 2007). A primary function of the AII amacrine, however, is to shift absolute sensitivity of the retina from scotopic (rod) vision to photopic (cone) vision, and vice versa. In humans, rods and cones share a common, ON-cone bipolar/ON-ganglion neuron pathway from the retina to the brain, and, depending upon the light level, the AII amacrine controls whether the visual centers receive scotopic or photopic information, or both at mesopic light levels (Field et al., 2009). In the revised model and discussed in more detail below, the AII amacrine neurons set the relatively high threshold of ipRGCs response to light through a rod-dominated shunting mechanism (Mitchell and Silver, 2003). For the model, as light levels increase, cones silence the rod response, thereby releasing the shunting inhibition of ipRGCs by the AII amacrine.

### Shunting Inhibition and the High Threshold for ipRGCs

The AII amacrine neurons form a tight network across the IPL, sending signals horizontally across the retina (Farsaii and Connaughton, 2007). These AII amacrine neurons have a bistratified morphology, meaning they have dendritic arbors in both the ON and the OFF sublayer of the IPL. Thus, the AII amacrine neurons form a processing network across the retina, controlling both ON and OFF signals from rod and cone bipolar neurons to ganglion cells exiting the eye and reaching the brain. ON-rod bipolar as well as ON-cone bipolar neurons synapse with this AII plexus (Process A in Figure 1) in the proximal, ON sublayer of the IPL (Farsaii and Connaughton, 2007). Under scotopic conditions, light stimulation of the rods activates the rod bipolar neurons that synapse with the AII amacrine cells. The AII amacrine cells then relay the scotopic signals to an ON-cone bipolar neuron which, in turn, synapses with an ON-ganglion neuron (Field et al., 2009; Demb and Singer, 2012). As light levels increase, the magnitude of neural signals from rods increase but cones also begin to respond to light. Under mesopic conditions both rods and cones signals are communicated through the H1 horizontal cell to the ON-cone bipolar/ON-ganglion pathway. As light levels continue to rise, the cone signals begin to dominate the ON-cone bipolar/ON-ganglion pathway because, in parallel, the cone signals suppress the rod signals through a bidirectional synapse with the AII amacrine (Trexler et al., 2001). The AII amacrine feeds back onto the rod bipolar, likely through a second amacrine (designated both as an AI and as an A17 amacrine) in the ON sublayer of the IPL, deactivating its input to the AII amacrine, and thus to the ON-cone bipolar/ON-ganglion pathway (Process A in Figure 1).

In addition to controlling rod and cone signaling to the ON-cone bipolar/ON-ganglion pathway, the AII amacrine network provides sign-inverting, OFF signals to other neurons through its distal dendritic arbor (Farsaii and Connaughton, 2007). This AII arbor in the OFF sublayer of the IPL spatially coexists with the M1 ipRGC dendritic tree in the most distal layer of the OFF sublayer of the IPL (Kolb, 2007, 2009). In the revised model, the AII arbor would provide the dominate rod response to electrically shunt the direct M1 ipRGC response to light, thereby elevating the threshold for light-induced stimulation of the SCN (Mitchell and Silver, 2003; O’Brien, 2014). This shunting inhibition would be created by tight synaptic junctions between the AII arbor and the M1 ipRGC dendrites, bleeding off the depolarizing current generated by photon absorption of the ipRGC neurons themselves (Process B in Figure 1). Do and Yau (2010) showed, for example, that M1 ipRGCs are prone to depolarizing blockades, particularly under dim conditions, consistent with the notion that shunting inhibition by rods, through the AII amacrine cells, controls their threshold. As light levels increase and cones become more and more active, the dominant rod response would be reduced and the shunting inhibition released enabling the M1 ipRGC to send electrical signals out of the retina to the SCN.

ON-cone bipolar neurons are of several types, depending upon the species (Petrides and Trexler, 2008). In humans, achromatic [V(λ)] as well as chromatic (spectrally opponent) bipolar neurons, including the parvocellular, midget (red ON vs. green OFF and green ON vs. red OFF), bipolar (Grünert, 1997) and the blue-ON bipolar (Demb and Singer, 2012) probably synapse with the AII amacrine network across the retina (Petrides and Trexler, 2008). In the revised model, as light levels increase, only two ON-cone bipolar (Demb and Singer, 2012) synapse with the AII amacrine. As shown in Figure 1, the AII amacrine receives, first, input from the achromatic V(λ), ON bipolar, reflecting the combined L- and M-cone input (+) to the H1 horizontal cell and, second, from the prevalent spectral opponent SB (e.g., Field et al., 2007), reflecting the spectral opponent S-cone/H2 horizontal cell input. Because the rod bipolar neurons and the modulating achromatic and SB neurons each have different spectral sensitivities, the spectrum and the amount of the light incident on the retina will affect the M1 ipRGC threshold in very complicated ways. Depending upon the relative stimulation of rods and L-, M-, and S-cones, the threshold for activating the ipRGC could vary for light sources that might appear the same. Therefore, the spectral irradiance distribution (both spectrum and amount) on the retina must be considered when modeling the threshold for circadian phototransduction.

It should be noted, however, that nocturnal mammals appear to have a different neurophysiological circuit giving them 3–4 orders of magnitude greater sensitivity to circadian-effective light (Bullough et al., 2006). Altimus et al. (2010) and Weng et al. (2013) showed that rods can directly stimulate the M1 ipRGC in the mouse, which would obviously imply a very low threshold to optical radiation. Østergaard et al. (2007) provided neuroanatomical support for this inference by showing that there is a direct synapse from the rod bipolar onto the ipRGC in rat. In contrast to the mouse and rat, rods do not have direct rod bipolar input into the ipRGC in diurnal humans, but again, for our species rods are postulated to elevate the threshold sensitivity of the ipRGC to direct stimulation by light through shunting inhibition by the AII amacrine.

### The A18 Amacrine

The AII amacrine neuron is the most widely studied, but there are at least 29 different amacrine cells (Kolb, 2007, 2009). The functions of these various amacrine cell types remain largely unknown. The revised model utilizes one of those incompletely understood amacrine neurons, the A18 amacrine. The A18 amacrine has features that would be consistent with the functionality of the original and revised models. First, the A18 is a dense and diffusely arborized neuron, with its dendrites largely in the most distal S1 region of the IPL OFF sublayer, the same location as the *en passant* synapses between the SB and the M1 ipRGC as well as with an unidentified dopaminergic amacrine (Dumitrescu et al., 2009; Kim et al., 2012). Second, the A18 is a dopaminergic, inhibitory neuron and is driven by the ON-pathway in the retina (Do and Yau, 2010), preferentially synapsing onto the AII amacrine (Kolb, 2007, 2009). In the revised model, this well-established connection serves to electrically uncouple the AII amacrine from the M1 ipRGC dendrites, releasing shunting inhibition (Process B in Figure 1). For modeling purposes then, it was assumed that this unidentified amacrine is the A18 amacrine (Kolb, 2007, 2009).

### The Spectral “Notch” for Monochromatic Wavelengths

**FIGURE 2:**
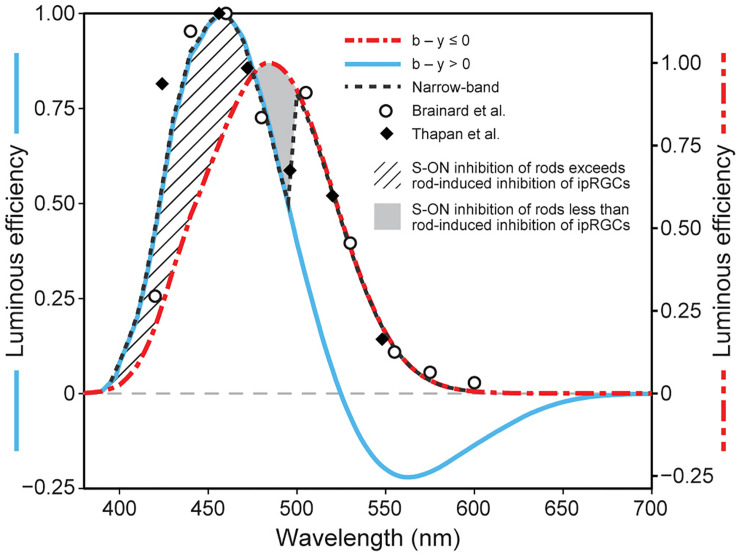
Model predictions of the spectral sensitivity of the circadian phototransduction circuit when exposed to either monochromatic (narrow-band) or polychromatic light sources at 300 scotopic lx at the eye. The spectral sensitivities to monochromatic spectral lights from two studies (closed diamonds Thapan et al., 2001; open circles Brainard et al., 2001) are shown together with the revised model predictions for monochromatic sources (black dashed line) and for polychromatic light sources where the b – y channel signals “blue” (blue solid line) or “yellow” (red dot/dash line). The cross-hatched area represents enhanced spectral sensitivity of the circadian phototransduction circuit to very short wavelengths (< 470 nm) when the SB signals “blue” and can provide added input to the ipRGC. The solid gray area represents the area of reduced spectral sensitivity, relative to that from the ipRGC alone (470–500 nm). This solid gray area of transition to longer wavelengths is due to a systematic loss of S-cone inhibition of rods as the b – y channel comes closer to its spectral cross-point at approximately 500 nm. As the inhibitory SB input to the AII amacrine is reduced (Process A in Figure 1), rod shunting inhibition of the ipRGC increases (Process B in Figure 1). For wavelengths longer than about 500 nm, the SB signals “yellow” OFF, and through the A18 amacrine neuron (Process C in Figure 1) decouples the shunting inhibition of the ipRGC altogether. It should be noted that the light level of 300 scotopic lx was chosen for illustration because rod inhibition is modeled to control threshold for ipRGC activation, thereby providing a common basis for comparing different spectral sensitivity functions and because, empirically, this light level is approximately equal to that producing the half-saturation response levels of nocturnal melatonin suppression. As described in the original model, a progressively smaller “notch” is predicted as light levels increase because cone inhibition of rods becomes relatively larger.

Single opsin action spectra exhibit a Gaussian-like sensitivity to different wavelengths with a half-bandwidth of approximately 80 nm. Further, photon absorption by these opsins is strictly additive for different combinations of wavelengths (principle of univariance; Rushton, 1972). In contrast, the spectral sensitivity of circadian phototransduction mechanism, as measured by nocturnal melatonin suppression by the pineal gland through the SCN, exhibits a half-bandwidth of approximately 100 nm and does not simply integrate flux at different wavelengths (Brainard et al., 2001; Thapan et al., 2001). Although the spectral sensitivity of the human SCN, as determined from experiments using light-induced nocturnal melatonin suppression, is similar to a Gaussian distribution, the derived spectral sensitivities to monochromatic lights from Brainard et al. (2001) and Thapan et al. (2001) both show a discontinuity at approximately 500 nm. When plotted together, a “notch” at this wavelength becomes more obvious (Figure 2). An explanation for this “notch” in the revised model is postulated to be through the A18 amacrine pathway and is essentially the same as that proposed originally in 2005.

For monochromatic sources, the sharp discontinuity occurs at 500 nm, near the cross-point from “blue” to “yellow” signals by the SB, b − y channel (Figure 2). Visually, this wavelength would correspond to unique green (Welbourne et al., 2013). For wavelengths shorter than 500 nm, it is postulated that the ipRGCs signal would reflect both its intrinsic photosensitive response and the SB input through the *en passant* synapses (Dumitrescu et al., 2009). For these short wavelengths, rod shunting inhibition also would be reduced by the SB input to the AII amacrine plexus (Process A in Figure 1; Demb and Singer, 2012). For very short wavelengths (e.g., 460 nm), the relatively strong SB input to the ipRGC along with the reduction in the rod shunting inhibition would, overall, enhance sensitivity of the circadian phototransduction mechanism relative to the M1 ipRGC (melanopsin) alone (cross-hatched area in Figure 2). So, for these very short wavelengths the M1 ipRGC and the SB responses determine spectral sensitivity. As monochromatic wavelength stimuli become longer (e.g., 490 nm), approaching the cross point of the b − y channel, the relative strength of the blue-ON signal from the SB would be diminished, further increasing shunting inhibition, and thereby reducing the sensitivity of circadian phototransduction in this region of short-wavelength light relative to M1 ipRGC (melanopsin) alone (solid gray area in Figure 2). At approximately 500 nm, the cross point of the b − y color signal, the SB would cease providing excitatory input to the M1 ipRGC in the model and, importantly, rod shunting inhibition would be released by the “yellow” OFF response of the spectrally opponent SB (Process C in Figure 1) through the dopaminergic A18 amacrine (Process B in Figure 1; Kolb, 2007; Dumitrescu et al., 2009; Kolb, 2009). The shift to wavelengths longer than the b − y cross point would suddenly increase the sensitivity of the circadian phototransduction mechanism, returning the spectral sensitivity to that of the M1 ipRGC characterized by melanopsin only. As in the original model the A18 amacrine serves as a diode. It can only signal the “yellow” OFF response from the spectrally opponent SB, which then decouples the shunting inhibition of the M1 ipRGCs by the AII amacrine.

These postulated amacrine mechanisms (Processes A–C in Figure 1) controlling rod shunting inhibition were part of the original model and were retained in the revised model. The neural foundation of the revised model was reinforced by new findings in neuroanatomy and neurophysiology.

### Maintaining the ON and OFF Pathways

As already described, it was postulated that the M1 ipRGC receives depolarizing responses from spectrally opponent SB neurons. The newly discovered, unusual *en passant* synapses with the SB ON response in the most distal (S1) OFF sublayer is a key element in the revised model. In this way, the SB ON response can combine with the intrinsic ON response of the M1 ipRGC. Implicit with *en passant* connections, however, the strict separation between ON and OFF pathways exiting the eye (Nelson and Connaughton, 2012) may be compromised by the *en passant* synapses unless there is some clear way to segregate the ON (blue) information from the OFF (yellow) information that is also carried by the spectral opponent SB. A recently described S-cone amacrine (SCA) that forms conventional synapses with the SB in the most proximal (S5) ON sublayer of the IPL (Patterson et al., 2020) may help maintain the clear separation between the ON and OFF pathways.

Among the many functions that amacrine cells perform, they often reverse the sign of the neural signals that reach them converting, for example, ON afferent input to OFF efferent output (Chen and Li, 2012). A spectral opponent, b − y amacrine cell type in the retina had already been described (Chen and Li, 2012), but Patterson et al. (2020) have provided new important details on the neuroanatomy of a spectrally opponent SCA as it potentially interacts with the SB and the M1 ipRGC.

Although Patterson et al. (2020) did not provide electrophysiological data to accompany their neuroanatomical findings, in the revised model, this specialized amacrine would invert the blue-ON/yellow-OFF signal from the SB, to a blue-OFF/yellow-ON inhibitory feedback signal to the SB, presynaptic to the ipRGC as suggested by Chen and Li (2012). By this assumed feedback, it is postulated that a light evoking an excitatory “blue” signal from the spectral opponent SB to the ipRGC would be reduced by an inhibitory blue-OFF signal generated by the SCA back onto the SB. When a light evokes a yellow-OFF response from the spectral opponent SB, the SCA completely counteracts that yellow-OFF signal with its yellow-ON signal. Generally, excitatory signals from a neuron are of greater magnitude than inhibitory signals. Thus, the SCA would generate a smaller, inhibitory blue-OFF signal to counteract the larger blue-ON signal from the SB and when the SB generates a smaller yellow-OFF signal, the SCA would produce a larger, yellow-ON signal. In this way the “blue” ON signal from the SB is merely reduced while the “yellow” OFF signal from the SB is completely eliminated. The M1 ipRGC therefore can only accept and then transmit to the SCN the blue-ON signal from the SB. As indirect support for this proposition, M1 ipRGC neurons (unlike M2 ipRGC neurons) are not spectrally opponent, conducting only ON responses to the SCN (Zhao et al., 2014; Graham and Wong, 2016). Thus, this SCA feedback mechanism postulated here would help preserve the strictly parallel ON and OFF pathways that might otherwise have been compromised by the *en passant* ON synapse in the OFF sublayer of the IPL. As a speculative, but consistent suggestion with that offered here for the SCA, the A18 amacrine may play a similar role of maintaining the strictly ON pathway for the M1d ipRGCs.

## Summary

Real progress has been made in the last 15 years regarding our collective understanding of circadian phototransduction. New insights have been gained into retinal neurophysiology and neuroanatomy and additional psychophysical data relating physical aspects of the stimulus to nocturnal melatonin suppression have been published. As discussed in the companion paper, some of these psychophysical experiments were designed to challenge the 2005 model of circadian phototransduction through *a priori* hypothesis testing. Synthesizing these new sources of information provided a more detailed framework for conceptualizing and modeling circadian phototransduction. However, two significant limitations of the current model described here and in the companion paper are the temporal dynamics of the phototransduction circuitry and the distribution of circuitry across the retina. The companion paper begins to address these limitations, but the temporal and spatial domains demand a great deal more study where both the retinal neurophysiology and the psychophysics of circadian phototransduction are addressed.

Much of the neuroanatomy and neurophysiology postulated to underlie the 2005 model remain unchanged for the revised model (Figure 1). The ipRGCs do not act alone in circadian phototransduction but, rather, more distal retinal neural processing is also involved. In particular, the SB neurons were conceptualized as providing input to the M1 ipRGC if the spectral composition of the light source produced a “blue” signal from this spectral opponent bipolar. Recent findings now show that *en passant* connections between the SB neurons and the M1 ipRGCs in the distal sublayer of the IPL provide the neuroanatomical foundation for this aspect of the revised model.

As in the 2005 model the revised model includes a dopaminergic A18 amacrine that helps define the spectral sensitivity of ipRGC for “warm” spectra. When the SB signals “yellow,” the A18 completely disconnects the shunting inhibition of the ipRGC by the AII amacrine, which is illustrated in Figure 2 (solid gray area) for wavelengths slightly shorter than the cross-point of the spectrally opponent b − y channel (circa 500 nm). The primary efferent connections of the A18 are with the AII amacrine (Process B in Figure 1) and new findings (Dumitrescu et al., 2009; Kim et al., 2012) suggest that the A18 probably receives afferent input from the *en passant* complex of connections with the SB and the ipRGC (Process C in Figure 1).

New to the revised model is the threshold term for the M1 ipRGC. As new psychophysical data were obtained, it became clear that the sensitivity to “warm” (b – y ≤ 0) sources was commonly overestimated relative to “cool” sources (b – y > 0) in the 2005 model (Nagare et al., 2019). By including an orthodox rod-cone mechanism to control shunting inhibition of M1 ipRGCs by the AII amacrine (Process A in Figure 1), it was possible to better align the data from “warm” and “cool” sources. In the revised model, this threshold mechanism is obviated when light levels are in the photopic range and the SB signals “yellow” because the A18 will decouple the shunting inhibition of the M1 ipRGCs from the AII amacrine. In other words, when light levels are high enough for cones to drive the SB and the balance of photic stimulation is on the “warm” side of the b − y channel, the ipRGCs alone perform circadian phototransduction.

## Data Availability Statement

The raw data supporting the conclusions of this article will be made available by the authors, without undue reservation.

## Author Contributions

MR conceived and developed the model and served as the primary author of the manuscript. RN provided the preliminary model framework, and contributed to the manuscript. MF supervised model synthesis, and provided leadership in preparation of the manuscript. All authors contributed to the article and approved the submitted version.

## Conflict of Interest

The authors declare that the research was conducted in the absence of any commercial or financial relationships that could be construed as a potential conflict of interest.
